# Supplementation with Natto and Red Yeast Rice Alters Gene Expressions in Cholesterol Metabolism Pathways in ApoE^-/-^ Mice with Concurrent Changes in Gut Microbiota

**DOI:** 10.3390/nu15040973

**Published:** 2023-02-15

**Authors:** Haiyan Zhou, Wenjing Liu, Yiqian Lv, Ke Liu, Yin Wang, Shuangli Meng, Tong Kang, Yuechao Bao, Huicui Meng

**Affiliations:** 1 School of Public Health (Shenzhen), Shenzhen Campus of Sun Yat-sen University, Shenzhen 518107, China; 2 School of Public Health (Shenzhen), Sun Yat-sen University, Guangzhou 510006, China; 3 School of Public Health, Baotou Medical College, Baotou 014040, China; 4 Guangdong Provincial Key Laboratory of Food, Nutrition and Health, Guangzhou 510080, China; 5 Guangdong Province Engineering Laboratory for Nutrition Translation, Guangzhou 510080, China

**Keywords:** natto, red yeast rice, gene expression, cholesterol metabolism, gut microbiota

## Abstract

We aimed to examine the effect of natto and red yeast rice (NR) supplementation on lipid and lipoprotein profiles, gene expressions of cholesterol metabolism, and the composition of gut microbiota in ApoE^-/-^ mice. Forty-one male ApoE^-/-^ mice aged 7–8 wks old were randomly fed a control diet (CD), CD + NR (oral gavage at 0.3 g/kg BW/day), high-fat and high-cholesterol diet (HFD), or HFD + NR for 12 wks. Fasting blood samples, liver and intestine tissues and fecal samples were collected at week 12. Biochemical parameters, gene expressions in cholesterol metabolism and gut microbiota composition and diversity were measured using standard methods. NR supplementation had no significant effect on lipid and lipoprotein profiles. Compared with the HFD group, HFD + NR resulted in higher mRNA expressions of HMGCR and CYP7A1 (both *P-NR* < 0.05) and ABCA1 (P-diet*NR = 0.0134, *P-NR* = 0.0407), lower mRNA expression of PCSK9 (P-diet*NR = 0.0002), lower fasting glucose concentrations (P-diet*NR = 0.0011), and lower relative abundance of genera *Bacteroides* and *Lactococcus* (both *P-NR* < 0.01) and *Coriobacteriaceae_UCG-002* (P-diet*NR = 0.0007). The relative abundance of *Lactococcus* was inversely correlated with HMGCR and CYP7A1, and the relative abundance of *Coriobacteriaceae_UCG-002* was positively correlated with PCSK9 and inversely correlated with ABCA1 (all *P* < 0.05). These findings suggest that NR supplementation may regulate gene expressions in cholesterol metabolism via changes in the gut microbiota in HFD-fed ApoE^-/-^ mice.

## 1. Introduction

Cardiovascular disease is the leading cause of morbidity and mortality globally [1]. Hypercholesterolemia is among the top risk factors for the development of atherosclerotic cardiovascular disease (ASCVD) [2], and strategies aimed at lowering circulating total cholesterol (TC) and low-density lipoprotein-cholesterol (LDL-C) can delay or eliminate atherosclerotic lesions and reduce the incidence of ASCVD and related death [3]. Statins, such as atorvastatin and lovastatin, are commonly used drugs to treat hypercholesterolemia. However, the long-term administration of these synthetic lipid-lowering drugs has been reported to trigger adverse effects or contraindications, such as liver dysfunction [4]. Therefore, adherence to a healthy lifestyle, including following healthy dietary patterns and eating behaviors and being physically active, has been proposed in many guidelines for lipid management and ASCVD prevention [5].

Several functional foods enriched in bioactive components have been reported to have cholesterol-lowering properties [6] and may be used as supplements for lipid management. Natto, a fermented food mainly consumed in Asian countries, is produced from soybeans through fermentation by *B. subtilis natto* [7]. Nattokinase (NK), which is the most active ingredient in natto, is a potent fibrinolytic enzyme demonstrating beneficial effects on cardiovascular health [8,9]. Previous studies have reported that the consumption of natto for 2–7 weeks reduces circulating TC or LDL-C concentrations in both human subjects [10] or experimental animals [11,12]. However, the mechanism underlying the cholesterol-lowering effect of natto has not been reported. Red yeast rice (RYR), which is also a fermented food mainly consumed in Asian countries, is produced by the fermentation of rice by *Monascus purpureus Went*, a species of the mound [13,14]. RYR contains a bioactive substance called monacolin K (lovastatin), which inhibits 3-hydroxyl-3-methylglutaryl coenzyme A (HMG-CoA) reductase in the pathway of cholesterol synthesis together with other monacolins in RYR [6]. Furthermore, RYR contains phytosterols, fiber and niacin, which collectively may exert cholesterol-lowering effects [15]. Several studies, including animal studies, randomized controlled trials and meta-analyses, have reported the beneficial effects of RYR on reducing circulating TC or LDL-C concentrations [13,16,17,18,19,20]. In addition, RYR and its bioactive components have been reported to downregulate the gene expressions of HMG-CoA reductase [19] and Niemann-Pick-C1-Like 1 (NPC1L1) [20] and upregulate the gene expressions of LDL receptor (LDLR) and Cholesterol 7-alpha hydroxylase (CYP7A1) [13,20], indicating that they may decrease circulating TC and LDL-C concentrations via the inhibition of cholesterol synthesis and the absorption and promotion of hepatic cholesterol uptake and bile acid synthesis.

In addition to the traditional mechanisms regulating cholesterol metabolism, gut microbiota has been reported to influence circulating cholesterol concentrations through their role in bile acid metabolism and the generation of microbial metabolites, such as short-chain fatty acids (SCFAs) and trimethylamine-N-oxide (TMAO) [21,22,23,24]. Both natto and RYR are fermented by food microorganisms, and potential interactions between food and gut microbes may also have an impact on health. An animal study has reported that RYR supplementation results in concurrent reductions in plasma TC and LDL-C concentrations and the relative abundance of fecal *Alistipes* and *Flavonifractor* [19]. In addition, the plasma TC and LDL-C concentrations have positive correlations with the relative abundance of these two gut bacteria, indicating that changes in gut microbiota may partially contribute to the cholesterol-lowering effect of RYR [19]. The effects of natto consumption on the composition and diversity of gut microbiota have not been reported and require further investigation.

Although existing studies have demonstrated the cholesterol-lowering effects of natto or RYR alone, the synergistic effects of supplementation with both natto and RYR on cholesterol metabolism have not been fully investigated. To, only one randomized controlled trial has reported the cholesterol-lowering effect of combined supplementation with both nattokinase and RYR rather than nattokinase alone [25]. However, the potential underlying mechanism is still unclear. The aim of the current study was to investigate the effect of supplementation with both natto and RYR on circulating cholesterol concentrations and potential underlying mechanisms, including gene expressions involved in cholesterol metabolism and the diversity and composition of gut microbiota and microbial metabolites.

## 2. Materials and Methods

### 2.1. Animals and Experimental Diets

The animal study was conducted according to established guidelines and protocols approved by the Sun Yat-sen University Animal Care and Use Committee (approval no. SYSU-IACUC-2021-000228). Because there are extremely limited published data on the effects of supplementation with both natto and RYR on circulating cholesterol concentrations, gene expressions in cholesterol metabolism and changes in gut microbiota, we based our sample size estimation on a previously established method called “resource equation” [26]. According to this method, the difference between the total number of animals and the total number of groups should lie between 10 and 20. Based on our study design, the total number of animals for four groups was between 14 (n = 4 per group) to 24 (n = 6 per group), and n = 10–11 per group was used in the current experiment. A total of 41 male ApoE^-/-^ mice of 6–7 weeks of age were purchased from the Beijing Vital River Laboratory Animal Technology Co., Ltd. (Beijing, China) and were housed in ventilated cages on 12 h light/dark cycle with access to food and water ad libitum in the animal facility of Sun Yat-sen University (Guangzhou, China). After acclimation to the normal chow diet for 7 days, mice were randomly allocated to four diet groups according to a 2 × 2 factorial design: (1) control diet (CD, MD12014, 12% kcal from fat, n = 10); (2) CD plus oral gavage with 0.3 g/kg BW/day solution made from natto red yeast rice (NR) Capsules (CD + NR, n = 11); (3) high-fat and high-cholesterol diet (HFD, MD12015, 41% kcal from fat, 0.15% cholesterol, n = 10), and (4) HFD plus oral gavage with 0.3 g/kg BW/day NR (HFD + NR, n = 10). Diets were purchased from Medison Biomedical Co., Ltd. (Yangzhou, Jiangsu, China) and NR capsules were purchased from Weihai Nanbowan Biotechnology Co., Ltd. (Weihai, Shandong, China). The main components of NR were natto and red yeast rice, and the content of monacolin K (lovastatin) was 0.2 g in 100 g of NR. The NR powders isolated from capsules were dissolved 3:100 (*w/v*) with a sterile phosphate-buffered saline solution (Corning, Corning, NY, USA), and an NR suspension or sterile phosphate-buffered saline was administered once daily (100 µL/10 g BW) by oral gavage. The food intakes and body weights were monitored and recorded weekly. At the end of the 12-week treatment, mice were fasted for 14 h and were then anesthetized and euthanized. Fasting blood samples were collected in anticoagulation tubes, and plasma samples were isolated following centrifugation at 10,000× *g* for 10 min at 4 °C. The liver and intestine samples and entire hearts with the aorta were carefully harvested and rinsed. Liver samples were dissected into smaller pieces and snap-frozen in liquid nitrogen and stored at −80 °C for subsequent analysis. After removal of the intestinal contents, the entire intestine was rinsed with ice-cold 1 × phosphate-buffered saline (pH 7.4) (Servicebio, Wuhan, Hubei, China), cut longitudinally and spread out on a glass plate on top of ice. The opened intestines were gently scraped with glass microscope slides, and intestinal mucosa was collected and stored at −80 °C for the subsequent analysis with a Real-Time quantitative Polymerase Chain Reaction (RT-qPCR). The aorta was isolated from the pulmonary trunk and the base of the heart, and the heart and aorta were preserved in 4% paraformaldehyde for no less than 48 h for further histologic measurement.

### 2.2. Biochemical Measurement of Blood Samples

Fasting blood glucose concentrations were measured by ACCU-CHEK^®^ Performa (Roche, Basel, Switzerland, Germany). Plasma concentrations of cardiometabolic risk factors, including triglycerides (TG), total cholesterol (TC), HDL-cholesterol (HDL-C), and LDL-cholesterol (LDL-C) were measured using a 3100 automatic biochemistry analyzer (Hitachi, Tokyo, Japan), with enzymatic or immunoturbidimetric reagents as per the manufacturer’s protocols. Plasma concentrations of VLDL-cholesterol (VLDL-C) were calculated as VLDL-C = TG/2.2 [27], and non-HDL-cholesterol (non-HDL-C) was calculated as non-HDL-C = TC − HDL-C [28]. Plasma PCSK9 concentrations were determined with an ELISA kit (Sino Biological, Beijing, China) according to the manufacturer’s protocol.

### 2.3. Atherosclerotic Lesion Measurement

Lipid deposition and atherosclerotic lesions of the aorta were determined with Oil Red O staining. Briefly, fixed aortas were cut longitudinally along the vessel wall with iris scissors and were stained with 60% (*w/v*) Oil Red O staining solution (Servicebio) at 37 °C for 60 min. After staining, excessive Oil Red O solutions were removed by washing the stained aortas with 15 mL 75% ethanol until the fatty plaques in the lumen of aortas became orange or bright red while the other areas were relatively colorless. The stained aortas were then unfolded, flattened and spread out on a black plate, and digital images of the entire aorta were taken with a Nikon Eclipse microscope (Nikon, Tokyo, Japan). Stained areas were analyzed with Image J 1.53 software and expressed as the percentage of Oil Red O-positive areas in the total areas of aortas.

### 2.4. Gene Expression Analysis

A gene expression analysis was conducted according to methods in previous studies [29]. Briefly, total RNA was extracted from intestinal mucosa and liver with TRIzol reagent (Beyotime, Shanghai, China), and quantified with a Nanodrop 2000 spectrophotometer (Thermo Fisher Scientific, Waltham, MA, USA). Total RNA was then reverse transcribed into cDNA with the SweScript RT I First Strand cDNA Synthesis Kit (Servicebio) according to the manufacturer’s protocol. Gene expressions responsible for cholesterol synthesis (HMGCR and SREBP-2 in liver), cholesterol absorption (NPC1L1 and LXR-α in intestine), cholesterol uptake (LXR-α, LDLR, IDOL and PCSK9 in liver), cholesterol efflux (ABCG5, ABCG8 and ABCA1 in intestine), and bile acid synthesis (CYP7A1 and FXR in liver) were quantified with a three-step RT-qPCR method using SYBR Green mixture (Takara, Tokyo, Japan) and a Quant Studio™ 6 Flex Real-Time PCR system (Thermo Fisher Scientific). Sequences of all primers (Table S1) were designed according to previous studies and were synthesized by Sangon Biotech Co., Ltd. (Shanghai, China). The housekeeping gene glyceraldehyde-3-phosphate dehydrogenase (GAPDH) was used as the internal control. Gene expression was quantified using the 2^−ΔΔCT^ method and expressed as a fold change normalized to the expression in CD control mice.

### 2.5. Gut Microbiome Analysis

The total genomic DNA of fecal samples was extracted with the TIANamp Stool DNA Kit (Tiangen, Beijing, China) according to the manufacturer’s protocol. After evaluating the quality of DNA samples by NanoDrop and agarose gel electrophoresis, the extracted DNA samples were used to amplify the V3-V4 hypervariable region of the 16S rRNA gene with primers 341F (5′-CCTACGGGNGGCWGCAG-3′) and 805R (5′-GACTACHVGGGTAT-CTAATCC). The amplicons were then purified and quantified, and the pair-end library was conducted. High-throughput sequencing was performed with the Illumina NovaSeq PE250 library, and 16S rRNA sequencing was performed on an Illumina NovaSeq 6000 (Illumina, San Diego, CA, USA) platform.

### 2.6. Fecal SCFAs Measurement

Concentrations of SCFAs, including the acetic, propionic, isobutyric, butyric, isovaleric and valeric in fecal samples were measured as described previously [30]. Fecal samples (50 mg) were diluted 1:9 (*v/v*) with methanol (≥99.0% purity, Thermo Fisher Scientific) and homogenized on a vortex mixer. After centrifugation at 20,000 rpm at 4 °C for 15 min, the supernatant was mixed with 20 µL internal standard, including acetic acid-d4 (≥99.0% purity, CATO, Guangzhou, China), propionic acid-d5, butyric acid-d7, isovaleric acid-d7 and valeric acid-d9 (≥99.0% purity, MACKLIN, Shanghai, China), and the final volume equaled 100 µL with the methanol. Supernatant (50 µL) from the final volume was mixed with 3% (*v/v*) pyridine solution (50 µL, diluted with pure methanol, MACKLIN), 2-NPH·HCl (50 µL, 20 mmol/L, diluted with methanol, MACKLIN), EDC·HCl (50 µL, 250 mmol/L, diluted with pure water, Aladdin, Shanghai, China) [31]. After being mixed by a vortex mixer and water bath at 60 °C for 20 min, the mixtures were mixed with 15% (*w/v*) KOH (Aladdin) solution, and the water bath was continued for 20 min. When restored to room temperature, the mixtures were mixed with 1 mL of 0.5 mol/L phosphoric acid aqueous solution (Aladdin) and 1 mL of ether (CATO) and fully shaken for 3 min. After centrifugation at 3000 r/min for 10 min, the organic layer was taken and nitrogen-blown for initial mobility re-solubilization. The mixtures were transferred into autosampler vials and then analyzed on a liquid chromatography-triple quadrupole (TSQ Altis^TM^) tandem mass spectrometric (LC-TQ-MS) (Thermo Scientific) with an ACQUITY UPLC^®^ BEH C18 column (2.1 × 100 mm, 1.7 μm, Waters, Milford, MA, USA). Concentrations of SCFAs were quantified using the Thermo Xcalibur^TM^ 4.0 software.

### 2.7. Statistical Analysis

#### 2.7.1. 16S rRNA Sequencing Data Analysis

Raw sequencing data were subjected to low-quality filtering operations such as splicing, primer removal, and chimeras with Qiime 2 2020.2 software. According to the silva-132-99 database, amplicon sequence variants (ASVs) were classified via the corresponding species classification information, which was obtained by using the trained naive Bayes classifier. The α-diversity of gut microbiota was estimated by the indexes of gut microbial richness [Observe, Chao 1, Abundance-based Coverage Estimator (ACE)] and evenness (Shannon, Simpson, and J), which were calculated by R 4.0.3 software according to the ASVs table. A principal component analysis (PCA) was conducted using the weighted UniFrac distance matrices to estimate the microbial community clustering (β-diversity). The differences in the gut microbial compositions and community structures among the four dietary groups were assessed by ANOSIM analysis. Based on whether the data were under normal distribution, a two-way analysis of variance (ANOVA) or Scheirer-Ray-Hare test with the main effects of diet (HFD vs. CD, *P-diet*), supplementation with NR (yes vs. no, *P-NR*), and diet*NR interaction (P-diet*NR) was used to determine differences in the relative abundance of gut microbiota at different taxa levels ranging from phylum to species among four diet groups and followed by post-hoc analysis via the Tukey-Kramer or Kruskal-Wallis method, respectively. Furthermore, the Venn diagram was conducted using web analytics tools (accessed on 9 October 2021): http://www.bioinformatics.com.cn/. Graphs of the sequencing data were depicted with R 4.0.3. Statistical significance was considered at *P* < 0.05 for all statistical analyses including α- and β-diversity.

#### 2.7.2. Additional Statistical Analysis

All data were assessed for normality and equal variances prior to statistical analysis. In our study, diet and NR were the two main effect factors for analysis. For data under normal distribution, a two-way analysis of variance (ANOVA) with the main effects of diet (HFD vs. CD, *P-diet*) and supplementation with NR (yes vs. no, *P-NR*), and diet*NR interaction (P-diet*NR) was used to determine differences among groups, followed by multiple comparisons determined using the method as described previously [32]. If data were skewed, log or square root transformation was undertaken prior to analysis. For data that were not under normal distribution with or without transformation, a Scheirer-Ray-Hare test followed by a Kruskal-Wallis post hoc test was used for group comparisons. Correlations between the relative abundance of gut microbiota at phylum to genus levels with other parameters were conducted using a Pearson or Spearman correlation. Values that exceeded the mean by two standard deviations were identified as outliers and were excluded from the final analysis. All data were presented as mean ± standard error of the mean (SEM). All analyses were conducted and all figures were depicted with GraphPad Prism 9.0 software (GraphPad Software; La Jolla, CA, USA) and IBM SPSS Statistics 26.0 software (SPSS; Armonk, NY, USA). Statistical significance was accepted at the *P* < 0.05 level.

## 3. Results

### 3.1. Effect of Supplementation with NR on Weekly Food and Energy Intaks and Final Body Weights in CD- or HFD-Fed ApoE^-/-^ Mice

**Figure 1:**
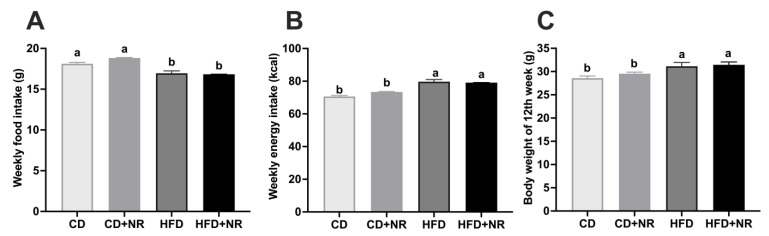
Weekly food and energy intakes and final body weights in ApoE^-/-^ mice fed with a CD or HFD, without or with NR at the end of the study. (**A**) Weekly food intake; (**B**) Weekly energy intake; (**C**) Final body weight at the end of the study. Data were presented as mean ± SEM. A statistical analysis was performed using a two-way analysis of variance (ANOVA) or a Scheirer-Ray-Hare test with the main effects of diet and NR and diet*NR interaction, depending on whether the data were under normal distribution. Means with different letters (a or b) indicated a significant difference from each other. CD (control), n = 10; CD + NR, n = 11; HFD, n = 10; HFD + NR, n = 10. CD, control diet; HFD, high-fat and high-cholesterol diet; + NR, a diet plus oral gavage with 0.3 g/kg BW/day of NR.

During 12 weeks of treatment, although the weekly food intakes in ApoE^-/-^ mice were lower in HFD and HFD + NR than the CD and CD + NR groups (*P-diet* = 0.0183) (Figure 1A), the weekly energy intakes were higher in HFD and HFD + NR than CD and CD + NR groups due to the higher energy density of HFD (*P-diet* = 0.0058) (Figure 1B). Consequently, the body weights of ApoE^-/-^ mice fed with HFD and HFD + NR were significantly higher than those fed with CD and CD + NR (*P-diet* = 0.0005) (Figure 1C). Supplementation with NR did not change food and energy intakes or body weights in mice fed with either CD or HFD (Figure 1A–C).

### 3.2. Effect of Supplementation with NR on Fasting Blood Concentrations of Biochemical Parameters and Atherosclerotic Lesion Development in CD- or HFD-Fed ApoE^-/-^ Mice

**Figure 2:**
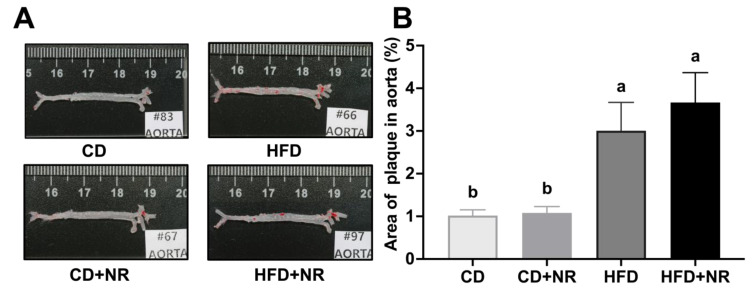
Histopathological changes of the entire aorta in ApoE^-/-^ mice fed a CD or HFD, without or with NR at the end of the study. (**A**) Oil Red O staining (×1 magnification); (**B**) Percentage of plaque area in aorta. Data were presented as mean ± SEM. A statistical analysis was performed using a two-way analysis of variance (ANOVA) with the main effects of diet and NR and diet*NR interaction. Means with different letters (a or b) indicated a significant difference from each other. CD (control), n = 10; CD + NR, n = 11; HFD, n = 10; HFD + NR, n = 10. CD, control diet; HFD, high-fat and high-cholesterol diet; + NR, a diet plus oral gavage with 0.3 g/kg BW/day of NR.

At the end of the 12-week treatment period, ApoE^-/-^ mice fed with HFD and HFD + NR resulted in higher fasting concentrations of plasma TC, HDL-C, non-HDL-C and PCSK9 and ratios of TC to HDL-C and LDL-C to HDL-C compared to mice fed with CD and CD + NR (all *P-diet* < 0.05) (Table 1). Supplementation with NR did not change these parameters, and there were no significant differences in TG, LDL-C and VLDL-C concentrations among the four groups. In comparison to HFD, HFD + NR resulted in lower fasting glucose concentrations in ApoE^-/-^ mice (P-diet*NR = 0.0011, *P-diet* < 0.0001) (Table 1). The percentage of atherosclerotic lesions as stained by Oil Red O of the entire aorta was higher in mice fed with HFD and HFD + NR in comparison to the mice fed with CD and CD + NR (*P-diet* < 0.0001) (Figure 2), with no significant effect from NR supplementation.

### 3.3. Effect of Supplementation with NR on Gene Expressions in Cholesterol Metabolism Pathways in CD- or HFD-Fed ApoE^-/-^ Mice

**Figure 3:**
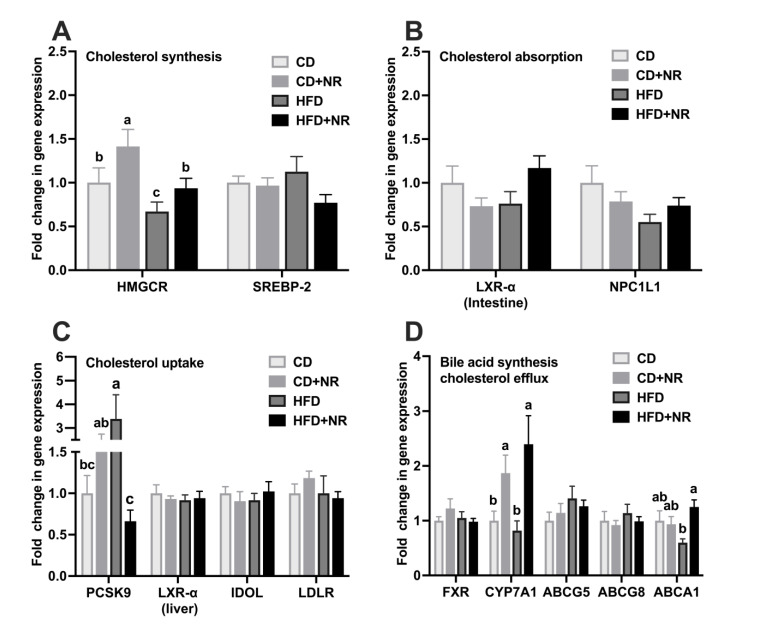
Gene expression of lipid metabolism pathways in ApoE^-/-^ mice fed a CD or HFD, without or with NR at the end of the study. (**A**) Gene expression in cholesterol synthesis; (**B**) Gene expression in cholesterol absorption; (**C**) Gene expression in cholesterol uptake; (**D**) Gene expression in cholesterol efflux and bile acid synthesis. Data were presented as mean ± SEM. A statistical analysis was performed using a two-way analysis of variance (ANOVA) with the main effects of diet and NR and diet*NR interaction. Means with different letters (a, b or c) indicate a significant difference from each other. CD (control), n = 10; CD + NR, n = 11; HFD, n = 10; HFD + NR, n = 10. ABCA1, Recombinant ATP Binding Cassette Transporter A1; ABCG5, ATP Binding Cassette subfamily G Member 5; ABCG8, ATP Binding Cassette Subfamily G Member 8; CD, control diet; CYP7A1, Cholesterol 7-alpha hydroxylase; FXR, Farnesoid X-activated receptor; GAPDH, glyceraldehyde-3-phosphate dehydrogenase; HFD, high-fat and high-cholesterol diet; HMGCR, Recombinant 3-hydroxy-3-methylglutaryl coenzyme a reductase; IDOL, Inducible degrader of the low-density lipoprotein receptor; LDLR, Low-density lipoprotein receptor; LXR-α, Recombinant liver X receptor Alpha; SREBP-2, Sterol regulatory element-binding protein-2; NPC1L1, Niemann-Pick C1-like 1; PCSK9, Proprotein convertase subtilisin/kexin type 9; + NR, a diet plus oral gavage with 0.3 g/kg BW/day of NR.

The hepatic mRNA expression of HMGCR, which is involved in cholesterol synthesis, was higher in the CD + NR and HFD + NR groups than the CD and HFD groups, respectively (*P-NR* = 0.0323, *P-diet* = 0.0123) (Figure 3A). The hepatic mRNA expression of PCSK9, which was involved in cholesterol uptake, was lower in the HFD + NR group than the HFD group (P-diet*NR = 0.0002) (Figure 3C). Compared to the CD and HFD groups, the hepatic mRNA expression of CYP7A1, which is involved in bile acid synthesis, was higher in the CD + NR and HFD + NR groups (*P-NR* = 0.0010) (Figure 3D). The intestinal mRNA expression of ABCA1, which is involved in cholesterol efflux, was higher in the HFD + NR than the HFD group (P-diet*NR = 0.0134, *P-NR* = 0.0407) (Figure 3D). There were no significant differences in the mRNA expression of other genes that are involved in cholesterol synthesis (hepatic SREBP-2), absorption (intestinal LXR-α and NPC1L1), uptake (hepatic LXR-α, IDOL and LDLR), or efflux (hepatic FXR and intestinal ABCG5 and ABCG8) pathways among four groups (Figure 3A–D).

### 3.4. Effect of Supplementation with NR on Gut Microbial Composition and Diversity in CD- or HFD-Fed ApoE^-/-^ Mice

**Figure 4:**
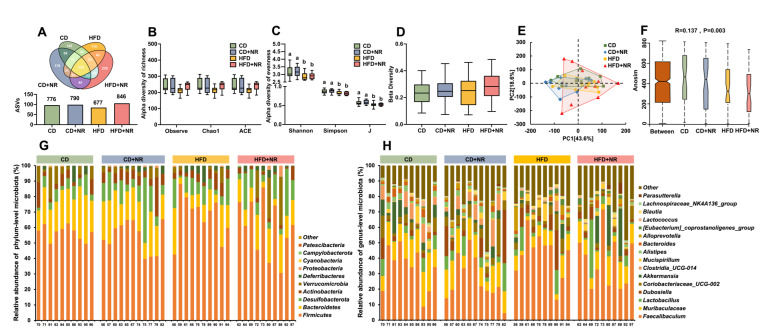
Gut microbial composition and diversity in ApoE^-/-^ mice fed with a CD or HFD, without or with NR at the end of the study. (**A**) Venn diagrams; (**B**) α-diversity of richness: Observe, Chao 1, ACE; (**C**) α-diversity of evenness: Shannon, Simpson, J indexes; (**D**) β-diversity; (**E**) Principal component analysis (PCA) based on ASVs level; (**F**) ANOSIM analysis diagrams; (**G**) Relative abundance of phylum-level microbiota; (**H**) Relative abundance of genus-level microbiota. Data were presented as mean ± SEM. A statistical analysis was performed using a two-way analysis of variance (ANOVA) with the main effects of diet and NR and diet*NR interaction. Means with different letters (a or b) indicated a significant difference from each other. CD (control), n = 10; CD + NR, n = 11; HFD, n = 10; HFD + NR, n = 10. CD, control diet; HFD, high-fat and high-cholesterol diet; + NR, a diet plus oral gavage with 0.3 g/kg BW/day of NR.

A total of 6,611,437 raw 16S rRNA reads from fecal samples of 41 mice were obtained in this study. After quality control filtering of 79,941–231,415 raw reads from each sample, we obtained 71,081–207,951 16S rRNA reads per sample for further analysis. Based on the Venn diagrams, both the CD and CD + NR groups had similar numbers of total ASVs, while there were 169 more ASVs in the HFD + NR group than in the HFD group. The number of group-specific ASVs was highest in the HFD + NR group, which was 27.8% of the total ASVs in this group, followed by 18.7%, 22.7% and 16.2% in the CD, CD + NR, and HFD groups, respectively (Figure 4A). There were no significant differences in the richness indexes of α-diversity among the four groups, including Observe, Chao 1 and ACE (Figure 4B). In contrast, the evenness indexes of α-diversity, including Shannon, Simpson and J, were significantly decreased in the HFD and HFD + NR groups compared with the CD and CD + NR groups (all *P-diet* < 0.05) (Figure 4C). The β-diversity of gut microbiota was similar among groups, and no distinct separation among the four groups was observed from the principal component analysis based on ASVs abundance (Figure 4D,E). An ANOSIM analysis with R > 0 indicated greater among-group differences than within-group differences, and it was necessary to make group comparisons (Figure 4F). The relative abundance of the top 10 dominant phyla and the top 16 dominant genera of all samples are shown in Figure 4G,H. *Firmicutes* (59.5%) and *Bacteroidetes* (17.6%) were the two most abundant phyla, which accounted for 77.1% of the total of all phyla (Figure 4G). *Faecalibaculum* (34.8%) and *Muribaculaceae* (12.1%) were the two most abundant genera, which accounted for 46.9% of all genera (Figure 4H).

### 3.5. Effect of Supplementation with NR on the Relative Abundance of Gut Microbiota at Different Taxa Levels and Concentrations of SCFAs in CD- or HFD-Fed ApoE^-/-^ Mice

At the phylum level, compared to the CD and CD + NR groups, both HFD and HFD + NR groups resulted in higher relative abundance of *Firmicutes* and lower relative abundance of *Bacteroidetes* and *Proteobacteria*, resulting in a higher ratio of *Firmicutes* to *Bacteroidetes* (all *P-diet* < 0.05) (Table 2). The relative abundance of these phyla did not change with NR supplementation. The relative abundance of *Actinobacteria* was lower in HFD + NR than HFD and CD + NR groups (P-diet*NR = 0.0016) (Table 2).

At the genus level, compared to the CD and CD + NR groups, both HFD and HFD + NR groups resulted in a higher relative abundance of the *Faecalibaculum* and *[Eubacterium]_coprostanoligenes_group*, and a lower relative abundance of the *Muribaculaceae*, *Alistipes*, *Alloprevotella* and *Lachnospiraceae_NK4A136_group* (all *P-diet* < 0.05) (Table 2). However, the relative abundance of these genera did not change with NR supplementation. The relative abundance of *Coriobacteriaceae_UCG-002* was lower in the HFD + NR than in the HFD and CD + NR groups (P-diet*NR = 0.0007) (Table 2). The relative abundance of *Bacteroides* in the HFD + NR and CD + NR groups were lower than those in the CD and HFD groups, respectively (*P-NR* = 0.0001, *P-diet* = 0.0006) (Table 2). Similarly, the relative abundance of *Lactococcus* was also lower in the CD + NR and HFD + NR groups compared to the CD and HFD groups (*P-NR* = 0.0013) (Table 2). The effect of supplementation with NR on the relative abundance of gut microbiota at the class, order, family and species levels in CD- or HFD-fed ApoE^-/-^ mice are summarized in Table S2.

In terms of SCFAs, both the HFD and HFD + NR groups resulted in lower concentrations of acetic acids and butyric acids in fecal samples than the CD and CD + NR groups (both *P-diet* < 0.05) (Table S3), and supplementation with NR did not alter these concentrations. CD + NR resulted in higher concentrations of propionic acids than the CD, HFD and HFD + NR groups (P-diet*NR = 0.0220, *P-diet* = 0.0357), and higher concentrations of isobutyric acids than the CD group (P-diet*NR = 0.0252, *P-NR* = 0.0342) (Table S3).

### 3.6. Correlations between the Relative Abundance of Gut Microbiota at Different Taxa Levels with Blood Biochemical Parameters, Gene Expressions in Cholesterol Metabolism, Atherosclerotic Lesions in the Aorta, and Fecal SCFAs Concentrations

**Figure 5:**
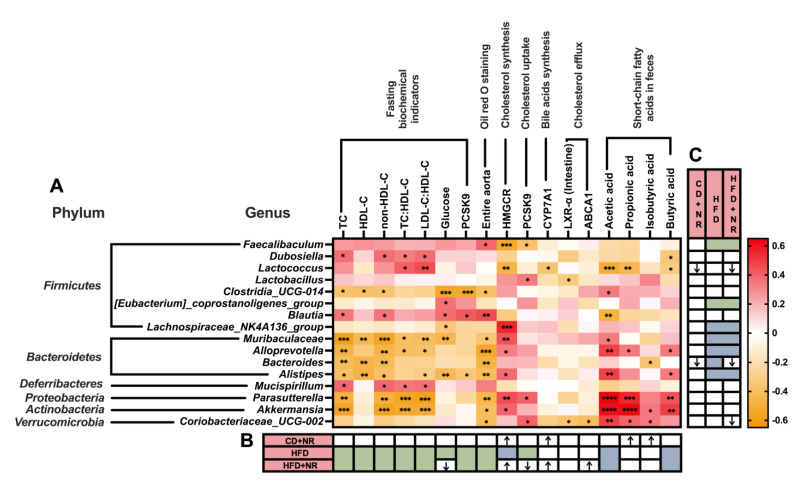
(**A**) Correlations between the relative abundance of gut microbiota (Top 16 genera) and blood biochemical parameters, Oil Red O staining of aorta, gene expressions in cholesterol metabolism and concentrations of SCFAs in ApoE^-/-^ mice fed with a CD and HFD, without or with NR at the end of the study. (**B**) Changes of corresponding parameters after different diet and NR interventions. (**C**) Changes in the relative abundance of gut microbiota after different diet and NR interventions. Pearson or Spearman correlation tests were used depending on the normality of the data (N = 41). The data were presented as Pearson or Spearman correlation coefficients. * *P* < 0.05, ** *P* < 0.01, *** *P* < 0.001, and **** *P* < 0.0001 indicate statistically significant correlations. CD (control), n = 10; CD+ NR, n = 11; HFD, n = 10; HFD + NR, n = 10. CD, control diet; HFD, high-fat and high-cholesterol diet; + NR, a diet with 0.3 g/kg BW/day supplementation of natto and red yeast. ↑ represented the parameters in the CD + NR group or the HFD + NR group that were significantly higher than those in the CD group or HFD group, respectively. ↓ represented the parameters in the CD + NR group or the HFD + NR group were significantly lower than those in CD group or HFD group, respectively. The color blue or green in the merged grid that represented the parameters in both HFD groups were significantly lower or higher than those in both CD groups. The color green in a single grid that represented the parameters in the HFD group were significantly higher than the CD group.

A total of 16 genera in 6 phyla levels had correlations with plasma biochemical parameters, hepatic and intestinal gene expressions in the cholesterol metabolism, atherosclerotic lesions in the aorta or SCFAs concentrations in fecal samples. The relative abundance of *Lactococcus*, which was lower in the CD + NR and HFD + NR groups than the CD and HFD groups, had positive correlations with fasting plasma TC: HDL-C and LDL-C: HDL-C ratios, and inverse correlations with the hepatic mRNA expression of HMGCR and CYP7A1 and fecal concentrations of acetic, propionic and butyric acids (all *P* < 0.05) (Figure 5). The relative abundance of *Bacteroides*, which was respectively lower in CD + NR and HFD + NR groups than the CD and HFD groups, had inverse correlations with fasting plasma concentrations of TC, HDL-C and non-HDL-C, and the percentage of atherosclerotic plaque in aorta and fecal concentrations of isobutyric acids (all *P* < 0.05) (Figure 5). The relative abundance of *Coriobacteraceae_UCG-002*, which was lower in the HFD + NR group than in the CD + NR and HFD groups, had positive correlations with the hepatic mRNA expression of PCSK9 and fecal concentrations of SCFAs, including acetic, propionic and isobutyric acids, and inverse correlations with the intestinal mRNA expressions of intestinal LXR-α and hepatic ABCA1 and the percentage of atherosclerotic plaque in the aorta (all *P* < 0.05) (Figure 5).

The relative abundance of the *Muribaculaceae*, *Alloprevtotalla*, *Alistipes* or *Lachnospirraceae_NKA136_group*, which were lower in both the HFD and HFD + NR groups compared to the CD and CD + NR groups, had positive correlations with the hepatic mRNA expression of HMGCR or fecal concentrations of acetic, propionic or butyric acids, and inverse correlations with fasting plasma concentrations of TC, HDL-C, non-HDL-C, glucose or TC: HDL-C and LDL-C: HDL-C ratios, and the percentage of atherosclerotic plaque in aorta or fecal concentrations of isobutyric acids (all *P* < 0.05) (Figure 5). The relative abundance of *Faecalibaculum*, which was higher in both the HFD and HFD + NR groups than the CD and CD + NR groups, had positive correlations with the percentage of atherosclerotic plaque in the aorta, and inverse correlations with the hepatic mRNA expressions of HMGCR and PCSK9 (all *P* < 0.05) (Figure 5). The relative abundance of the *[Eubacterium]_coprostanoligenes_group*, which was also higher in both the HFD and HFD + NR groups than the CD and CD + NR groups, had positive correlations with glucose concentrations (*P* < 0.05) (Figure 5). Correlations between the relative abundance of gut microbiota at the class, order, family and species levels with other parameters were summarized and are presented in Tables S4 and S5, respectively.

## 4. Discussion

In the current study, supplementation with NR resulted in lower fasting blood glucose concentrations in ApoE^-/-^ mice fed with HFD, with no significant effect on lipid and lipoprotein profiles. NR supplementation resulted in higher mRNA expressions of intestinal ABCA1 and hepatic CYP7A1, and the lower mRNA expression of hepatic PCSK9, concurrent with the lower relative abundance of phylum *Actinobacteria* and genera *Coriobacteriaceae_UCG-002*, *Bacteroides* and *Lactococcus* in ApoE^-/-^ mice fed with HFD. The relative abundance of *Coriobacteriaceae_UCG-002* was positively correlated with the hepatic mRNA expression of PCSK9 and inversely correlated with intestinal mRNA expression of ABCA1, and the relative abundance of *Lactococcus* was inversely correlated with the hepatic mRNA expression of CYP7A1. These findings collectively suggested that NR supplementation may regulate gene expressions in cholesterol metabolism via changes in the gut microbiota in ApoE^-/-^ mice fed with HFD.

Previous studies have reported the cholesterol-lowering effect of supplementation with natto or RYR alone in both human subjects and experimental animals [10,11,12,13,16,17,18,19,20]. However, in the current study, combined supplementation with NR at a dose of 0.3 g/kg BW/day for 12 weeks did not significantly change fasting plasma concentrations of TC, LDL-C, HDL-C or non-HDL-C. The reason for the discordant results may be attributed, in part, to the lower dose of NR used in the current study in comparison to the doses used in previous studies, including natto ranging from 1.0–2.0 g/kg BW/day [33,34,35] and red yeast rice ranging from 0.3–1.0 g/kg BW/day [19,36,37]. Of note, on the basis of body surface area, the dose of NR in the current study equaled 1.4 g for an adult with a standard body weight of 50 kg, which was equivalent to the recommended dose of commercially available NR supplements. Future studies are required to examine the dose-response of NR supplementation on lipid and lipoprotein profiles in order to find the optimal dose for the cholesterol-lowering effect.

Previous studies have reported the ability of RYR or monacolin K to downregulate the expression of HMGCR [19] or inhibit HMG-CoA reductase [6]. Inconsistent with these findings, we found that supplementation with NR resulted in higher mRNA expression of HMGCR in ApoE^-/-^ fed with either CD or HFD. The reason for the discordant results is not obvious. In the current study, the mRNA expression of HMGCR was associated with relative abundance of several gut bacteria, including a positive association with the *Muribaculaceae*, *Alloprevtotalla*, *Alistipes* and *Lachnospirraceae_NKA136_group* and inversely association with *Faecalibaculum*. In particular, the mRNA expression of HMGCR was inversely associated with the relative abundance of *Lactococcus*, which was lower in both NR-supplemented groups than the CD and HFD groups without supplementation. These findings indicated that NR supplementation may alter the gene expression of HMGCR via changes in the gut microbiota.

PCSK9 has been reported to inhibit the LDL receptor, resulting in lower cholesterol uptake by the liver and subsequent higher cholesterol concentrations in the circulation [38]. Intestinal ABCA1 promotes the efflux of free cholesterol from cells to HDL, which contributes to reverse cholesterol transport [39]. Based on our data, NR supplementation resulted in the lower mRNA expression of hepatic PCSK9 and higher mRNA expression of intestinal ABCA1 in ApoE^-/-^ mice fed with HFD than mice without supplementation. These results indicated that NR supplementation may promote cholesterol uptake and efflux via regulating the expression of genes involved in these pathways, although these alterations did not affect circulating cholesterol concentrations. In addition, the relative abundance of *Coriobacteriaceae_UCG-002*, which was lower following NR supplementation in ApoE^-/-^ mice fed with HFD than mice without supplementation, had a positive correlation with the hepatic mRNA expression of PCSK9 and an inverse correlation with the intestinal mRNA expression of ABCA1. Therefore, NR supplementation may regulate the gene expression of PCSK9 and ABCA1 via changes in the gut microbiota, which requires further investigation.

CYP7A1 is a key enzyme in the conversion of cholesterol to bile acids and plays an important role in maintaining the stability of cholesterol concentrations in the circulation [40]. Previous studies have reported that RYR and extracts upregulate the mRNA expression of CYP7A1 [13,20], which may contribute to the decrease of circulating cholesterol concentrations via promoting the conversion of cholesterol to bile acids. Our data were consistent with these findings, although we did not observe subsequent changes in plasma cholesterol concentrations after NR supplementation. The relative abundance of *Lactococcus*, which was lower in CD- or HFD-fed mice supplemented with NR in comparison to non-supplemented mice, had an inverse correlation with the hepatic mRNA expression of CYP7A1. Prior to our study, the effect of supplementation with natto or RYR or NR on the relative abundance of *Lactococcus* has not been reported. Further studies are required to explore whether NR supplementation could regulate the gene expression of CYP7A1 via changes in the gut microbiota.

The majority of previous studies focus on the effect of natto or RYR on lipid and lipoprotein profiles, and less attention has been paid to the glucose metabolism. The current study observed that NR supplementation resulted in lower fasting blood glucose concentration in ApoE^-/-^ mice fed with HFD, which was consistent with some prior reports with regard to RYR supplementation [41,42]. Recent studies have demonstrated the role of gut microbiota and related metabolites in the maintenance of glucose homeostasis [43]. Data from the current study also demonstrated that the relative abundance of the *Muribaculaceae*, *Alistipes* and *Lachnospirraceae_NKA136_group*, which were lower in both HFD and HFD + NR groups compared to CD and CD + NR groups, had inverse correlations with the fasting plasma concentrations of glucose. The relative abundance of the *[Eubacterium]_coprostanoligenes_group*, which was higher in both the HFD and HFD + NR groups compared to the CD and CD + NR groups, was positively correlated with fasting blood glucose concentrations. In accordance with our findings, previous studies have also reported changes in the relative abundance of *Muribaculaceae* [44,45], the *Lachnospiraceae_NK4A136_group* [46], and *Alistipes* [19], which is concurrent with changes in fasting glucose concentrations. Although NR supplementation did not significantly alter the relative abundance of these gut bacteria, their correlations with fasting blood glucose concentrations suggested that the gut microbiota may be partially attributable to the glucose-lowering effect of NR. However, the underlying mechanisms responsible for the glucose-lowering effect of NR are poorly understood and require further exploration.

Prior to the current study, there were no reports on the effect of NR supplementation on fecal concentrations of SCFAs. We found that NR supplementation resulted in higher concentrations of propionic acids and isobutyric acids in ApoE^-/-^ mice fed with CD. However, NR supplementation did not affect concentrations of SCFAs in ApoE^-/-^ mice fed with HFD, and hence the changes in mRNA expression of HMGCR, PCSK9, ABCA1 and CYP7A1 and fasting blood glucose concentrations in HFD-fed ApoE^-/-^ mice supplemented with NR may not be attributed to mediation via SCFAs. The fecal concentration of propionic acids was inversely correlated with the relative abundance of *Lactococcus*, which was lower in both NR-supplemented groups than in the CD and HFD groups without supplementation. Fecal concentrations of both propionic and isobutyric acids were positively correlated with the relative abundance of *Coriobacteriaceae_UCG-002*, which was lower in the HFD + NR than in the CD + NR and HFD groups, respectively. These findings indicated that *Coriobacteriaceae_UCG-002* may serve as a potential SCFA producer.

There are several limitations to the current study. Although the dose of NR supplementation was calculated to be equivalent with the recommended dose of commercially available NR supplements, the dose used in the current study was lower than those of previous studies, which may partially account for the lack of significant NR effect on lipid and lipoprotein profiles. We did not examine the dose-response of NR supplementation on lipid and lipoprotein profiles to find the optimal dose for the potential cholesterol-lowering effect. Replication of this experiment in another group of animals to confirm these findings is necessary in the future.

## 5. Conclusions

In summary, NR supplementation resulted in lower fasting blood glucose concentrations in ApoE^-/-^ mice fed with HFD. Although NR supplementation did not change plasma lipid and lipoprotein profiles, it may have the potential to promote cholesterol uptake, efflux and conversion to bile acids via upregulating the mRNA expressions of intestinal ABCA1 and hepatic CYP7A1 and downregulating the mRNA expression of hepatic PCSK9 in ApoE^-/-^ mice fed with HFD. Of note, concurrent with the changes in gene expression involved in cholesterol metabolism and glucose concentrations, the relative abundance of the phylum *Actinobacteria* and genus *Coriobacteriaceae_UCG-002* were lower in HFD-fed mice supplemented with NR, and the relative abundance of the genus *Bacteroides* and *Lactococcus* were lower in both the CD- and HFD-fed mice supplemented with NR. In agreement with these findings, the relative abundance of *Coriobacteriaceae_UCG-002* was positively correlated with the hepatic mRNA expression of PCSK9 and inversely correlated with the intestinal mRNA expression of ABCA1 and the relative abundance of *Lactococcus* was inversely correlated with the hepatic mRNA expression of CYP7A1. Collectively, NR supplementation may influence gene expressions in cholesterol metabolism via changes in the gut microbiota in ApoE^-/-^ mice fed with HFD, which requires further investigation.

## Figures and Tables

**Table 1:** Fasting blood concentrations of biochemical parameters in ApoE^-/-^ mice fed with a CD or HFD, without or with NR at the end of the study ^1^.

Variables	CD	CD + NR	HFD	HFD + NR	*P-Diet*	*P-NR*	*P-Diet* *NR
TC, mmol/L	22.5 ± 1.5 ^b^	24.9 ± 1.8 ^b^	40.2 ± 2.3 ^a^	37.0 ± 4.5 ^a^	<0.0001	0.89	0.33
HDL-C, mmol/L	1.0 ± 0.1 ^b^	1.1 ± 0.0 ^b^	1.3 ± 0.1 ^a^	1.4 ± 0.1 ^a^	<0.0001	0.07	0.97
non-HDL-C, mmol/L	21.5 ± 1.5 ^b^	23.7 ± 1.8 ^b^	38.8 ± 2.3 ^a^	35.6 ± 4.4 ^a^	<0.0001	0.87	0.33
TC: HDL-C	21.0 ± 2.1 ^b^	21.9 ± 1.3 ^b^	29.2 ± 2.9 ^a^	26.3 ± 3.0 ^a^	0.0139	0.68	0.44
LDL-C: HDL-C	7.1 ± 0.8 ^b^	7.6 ± 0.5 ^b^	10.4 ± 1.1 ^a^	9.0 ± 1.1 ^a^	0.0315	0.20	0.26
TG, mmol/L	1.2 ± 0.2	1.3 ± 0.2	1.6 ± 0.2	1.5 ± 0.1	0.21	0.87	0.76
LDL-C, mmol/L	7.6 ± 0.6	8.6 ± 0.7	14.4 ± 0.8	12.6 ± 1.6	0.24	0.34	0.40
VLDL-C, mmol/L	0.6 ± 0.1	0.6 ± 0.1	0.7 ± 0.1	0.7 ± 0.1	0.21	0.85	0.76
Glucose, mmol/L	4.3 ± 0.2 ^c^	5.0 ± 0.1 ^bc^	6.9 ± 0.5 ^a^	5.6 ± 0.2 ^b^	<0.0001	0.31	0.0011
PCSK9, ng/mL	3.1 ± 0.5 ^b^	3.0 ± 0.5 ^b^	12.1 ± 2.4 ^a^	16.0 ± 5.8 ^a^	<0.0001	0.74	0.77

^1^ Data were presented as mean ± SEM. A statistical analysis was performed using a two-way analysis of variance (ANOVA) with the main effects of diet and NR and diet*NR interaction. *P-diet* indicated significant main effect of diet (HFD vs. CD), *P-NR* indicated significant main effect of NR supplementation (NR supplementation vs. no supplementation), and P-diet*NR indicated significant interaction between diet and NR supplementation. Means with different letters (a, b or c) indicated a significant difference from each other. CD (control), n = 10; CD + NR, n = 11; HFD, n = 10; HFD + NR, n = 10. CD, control diet; HDL-C, HDL-cholesterol; HFD, high-fat and high-cholesterol diet; LDL-C, LDL-cholesterol; non-HDL-C, non-HDL-cholesterol; PCSK9, Proprotein convertase subtilisin/kexin type 9; TC, total cholesterol; TG, triglyceride; VLDL-C, VLDL-cholesterol; + NR, a diet plus oral gavage with 0.3 g/kg BW/day of NR.

**Table 2:** Relative abundance of phylum-level and genus-level gut microbiota in ApoE^-/-^ mice fed with a CD or HFD, without or with NR at the end of the study ^1^.

Gut Microbiota	CD	CD + NR	HFD	HFD + NR	*P-d* *iet*	*P-* *NR*	*P-d* *iet* *NR
Phylum							
*Firmicutes*	56.65 ± 1.48 ^b^	54.33 ± 3.04 ^b^	67.34 ± 4.49 ^a^	60.27 ± 5.84 ^a^	0.0451	0.25	0.56
*Bacteroidetes*	23.10 ± 1.91 ^a^	20.41 ± 1.78 ^a^	12.61 ± 2.94 ^b^	14.22 ± 2.35 ^b^	0.0008	0.81	0.35
*Desulfobacterota*	5.46 ± 0.81	9.95 ± 1.70	9.42 ± 1.79	9.82 ± 2.30	0.28	0.17	0.25
*Actinobacteria*	4.13 ± 1.02 ^ab^	5.87 ± 0.55 ^a^	5.40 ± 0.86 ^a^	2.08 ± 0.31 ^b^	0.10	0.29	0.0016
*Verrucomicrobia*	4.75 ± 1.42	2.07 ± 0.57	1.05 ± 0.44	7.60 ± 3.92	0.36	0.86	0.82
*Deferribacteres*	0.98 ± 0.57	1.20 ± 0.37	2.16 ± 0.63	2.10 ± 0.60	0.05	0.33	0.22
*Proteobacteria*	0.21 ± 0.05 ^a^	0.41 ± 0.10 ^a^	0.25 ± 0.11 ^b^	0.36 ± 0.29 ^b^	0.0476	0.66	0.30
*Firmicutes: Bacteroidetes*	2.43 ± 0.18 ^b^	2.91 ± 0.31 ^b^	9.78 ± 2.67 ^a^	5.47 ± 1.66 ^a^	0.0018	0.29	0.13
Genus							
*Faecalibaculum*	33.58 ± 4.40 ^b^	26.11 ± 4.31 ^b^	42.22 ± 4.63 ^a^	38.32 ± 5.49 ^a^	0.0332	0.24	0.71
*Muribaculaceae*	15.68 ± 1.59 ^a^	14.96 ± 1.01 ^a^	7.68 ± 1.24 ^b^	10.88 ± 1.63 ^b^	0.0001	0.38	0.17
*Lactobacillus*	4.01 ± 1.31	4.62 ± 1.22	4.80 ± 1.82	2.72 ± 0.72	0.83	0.89	0.25
*Dubosiella*	1.87 ± 0.43	4.76 ± 2.05	4.62 ± 1.35	2.28 ± 0.48	0.23	0.66	0.42
*Coriobacteriaceae_UCG-002*	3.46 ± 0.90 ^ab^	5.41 ± 0.55 ^a^	5.18 ± 0.85 ^a^	1.96 ± 0.28 ^b^	0.22	0.37	0.0007
*Akkermansia*	4.75 ± 1.42	2.07 ± 0.57	1.05 ± 0.44	7.60 ± 3.92	0.36	0.93	0.76
*Clostridia_UCG-014*	4.91 ± 1.58	4.37 ± 1.27	0.14 ± 0.01	0.28 ± 0.06	0.35	0.32	0.87
*Mucispirillum*	0.98 ± 0.57	1.20 ± 0.37	2.16 ± 0.63	2.10 ± 0.60	0.05	0.33	0.22
*Alistipes*	2.78 ± 0.44 ^a^	2.83 ± 0.40 ^a^	0.60 ± 0.23 ^b^	0.89 ± 0.26 ^b^	0.0229	0.73	0.90
*Bacteroides*	3.07 ± 0.43 ^a^	1.20 ± 0.23 ^b^	1.44 ± 0.30 ^b^	0.64 ± 0.15 ^c^	0.0006	0.0001	0.96
*Alloprevotella*	0.85 ± 0.12 ^a^	1.38 ± 0.34 ^a^	1.06 ± 0.68 ^b^	0.45 ± 0.29 ^b^	0.0006	0.51	0.23
*[Eubacterium]_coprostanoligenes_group*	0.31 ± 0.09 ^b^	0.20 ± 0.05 ^b^	1.77 ± 0.53 ^a^	0.67 ± 0.28 ^a^	0.0406	0.10	0.33
*Lactococcus*	0.82 ± 0.32 ^a^	0.35 ± 0.05 ^b^	0.88 ± 0.16 ^a^	0.33 ± 0.09 ^b^	0.98	0.0013	0.34
*Blautia*	0.23 ± 0.14	0.27 ± 0.13	0.96 ± 0.17	0.85 ± 0.26	0.05	0.81	0.59
*Lachnospiraceae_NK4A136_group*	0.75 ± 0.18 ^a^	0.70 ± 0.11 ^a^	0.24 ± 0.05 ^b^	0.54 ± 0.15 ^b^	0.0044	0.08	0.30
*Parasutterella*	0.18 ± 0.05	0.41 ± 0.10	0.24 ± 0.11	0.34 ± 0.29	0.23	0.85	0.48

^1^ Data are presented as mean ± SEM. A statistical analysis was performed using a two-way analysis of variance (ANOVA) or Scheirer-Ray-Hare test with the main effects of diet and NR and diet*NR interaction, depending on whether the data were under normal distribution. *P-diet* indicated the significant main effect of diet (HFD vs. CD), *P-NR* indicated the significant main effect of NR supplementation (NR supplementation vs. no supplementation), and P-diet*NR indicated the significant interaction between diet and NR supplementation. Means with different letters (a, b or c) indicated a significant difference from each other. CD (control), n = 10; CD + NR, n = 11; HFD, n = 10; HFD + NR, n = 10. CD, control diet; HFD, high-fat and high-cholesterol diet; + NR, a diet plus oral gavage with 0.3 g/kg BW/day of NR.

## Data Availability

Not applicable.

## Supplementary Materials

The following supporting information can be downloaded at: https://www.mdpi.com/article/10.3390/nu15040973/s1, Supplementary Table S1: Sequences of primers; Supplementary Table S2: Relative abundance of gut microbiota at class, order, family and species levels in ApoE^-/-^ mice fed with a CD or HFD, without or with NR at the end of the study; Supplementary Table S3: Fecal concentrations of short-chain fatty acids in ApoE^-/-^ mice fed with a CD or HFD, without or with NR at the end of the study; Supplementary Table S4: Correlations between relative abundance of gut microbiota and blood biochemical parameters, Oil Red O staining of aorta and gene expression in cholesterol metabolism; Supplementary Table S5: Correlations between the relative abundance of gut microbiota and concentrations of short-chain fatty acids in feces.
