# Supplementary material for: Supplementation with Natto and Red Yeast Rice Alters Gene Expressions in Cholesterol Metabolism Pathways in ApoE-/- Mice with Concurrent Changes in Gut Microbiota
Source: Nutrients. 2023 Feb 15;15(4):973. doi: 10.3390/nu15040973 (PMC9961320; doi:10.3390/nu15040973)
Supplement: Supplementary file 1 [file nutrients-15-00973-s001.zip › nutrients-2165956-supplementary.pdf]

Supplementary Table S1. Sequences of primers[1]

Gene Name	Forward primer (5′-3′)	Reverse primer (5′-3′)
GAPDH	GGTTGTCTCCTGCGACTTCA	TGGTCCAGGGTTTCTTACTCC
NPC1L1	CTGGCTGGCTCTCATCATCATCTTC	CTGCTGTCTTGTTCTTGTTCCTGTTG
IDOL	ATGCTGTGCTATGTGACGAGG	TCGATGATCCCTAGACGCCTG
LDLR	TGACTCAGACGAACAAGGCTG	ATCTAGGCAATCTCGGTCTCC
LXR-α	GAGTGTCGACTTCGCAAATG	CTTCAGTTTCTTCAAGCGGATC
PCSK9	AGCAGCCAGGTGGAGGTGTATC	CTTGCTCGCCTGTCTGTGGAAG
HMGCR	GGTGCAAAGTTCCTTAGTGATG	GAATAGACACACCACGTTCATG
CYP7A1	GTGATGTTTGAAGCCGGATATC	TTTATGTGCGGTCTTGAACAAG
SREBP-2	TTTTACTGAAGTAGAGCGGGTC	CATGCATGGCTCTACAGGTATA
FXR	GCAACCAGTCATGTACAGATTC	TTATTGAAAATCTCCGCCGAAC
ABCA1	CCTCAGAGAAAACAGAAAACCG	CTTTGCTATGATCTGCACGTAC
ABCG5	CATTGAAAGAGCACGATACCTG	AGATTCTGAACGAGACGCATAA
ABCG8	GGACAAATTTGGATAAATGGGC	GATTACGTCTTCCACCCGTTT

[1]ABCA1, Recombinant ATP Binding Cassette Transporter A1; ABCG5, ATP Binding Cassette subfamily G Member 5; ABCG8, ATP Binding Cassette Subfamily G Member 8; CYP7A1, Cholesterol 7-alpha hydroxylase; FXR, Farnesoid X-activated receptor; GAPDH, glyceraldehyde-3-phosphate dehydrogenase; HMGCR, Recombinant 3-hydroxy-3-methylglutaryl coenzyme a reductase; IDOL, Inducible degrader of the low-density lipoprotein receptor; LDLR, Low-density lipoprotein receptor; LXR-α, Recombinant liver X receptor Alpha; SREBP-2, Sterol regulatory element-binding protein-2; NPC1L1, Niemann-Pick C1-like 1; PCSK9, Proprotein convertase subtilisin/kexin type 9.

Supplementary Table S2. Relative abundance of gut microbiota at class, order, family and species levels in ApoE$^{-/-}$ mice fed with a CD or HFD, without or with NR at the end of the study[1]

Gut microbiota	CD	CD + NR	HFD	HFD + NR	*P-diet*	*P-NR*	*P-diet*NR*
**Class**							
Bacilli	43.22 ± 2.67^{b}	40.45 ± 3.86^{b}	55.99 ± 4.46^{a}	48.83 ± 5.82^{a}	0.0197	0.26	0.62
Bacteroidia	23.10 ± 1.91^{a}	20.41 ± 1.78^{a}	12.61 ± 2.94^{b}	14.22 ± 2.35^{b}	0.0008	0.81	0.35
Clostridia	13.43 ± 2.03	13.88 ± 1.42	11.35 ± 1.54	11.44 ± 1.35	0.17	0.87	0.91
Desulfovibrionia	5.46 ± 0.81	9.95 ± 1.70	9.42 ± 1.79	9.82 ± 2.30	0.28	0.17	0.25
Coriobacteriia	3.52 ± 0.90^{ab}	5.45 ± 0.55^{a}	5.22 ± 0.85^{a}	1.99 ± 0.28^{b}	0.22	0.36	0.0008
Verrucomicrobiae	4.75 ± 1.42	2.07 ± 0.57	1.05 ± 0.44	7.60 ± 3.92	0.36	0.93	0.76
Deferribacteres	0.98 ± 0.57	1.20 ± 0.37	2.16 ± 0.63	2.10 ± 0.60	0.05	0.33	0.22
Gammaproteobacteria	0.20 ± 0.05	0.41 ± 0.10	0.25 ± 0.11	0.36 ± 0.29	0.05	0.62	0.28
Actinobacteria	0.74 ± 0.24^{a}	0.42 ± 0.12^{b}	0.18 ± 0.03^{b}	0.06 ± 0.03^{c}	<0.0001	0.0182	0.21
**Order**							
Erysipelotrichales	36.67 ± 4.01^{b}	33.62 ± 3.21^{b}	49.05 ± 4.01^{a}	41.29 ± 5.39^{a}	0.0219	0.20	0.58
Bacteroidales	23.10 ± 1.91^{a}	20.41 ± 1.78^{a}	12.61 ± 2.94^{b}	14.22 ± 2.35^{b}	0.0008	0.81	0.35
Desulfovibrionales	5.46 ± 0.81	9.95 ± 1.70	9.42 ± 1.79	9.82 ± 2.30	0.28	0.17	0.25
Lactobacillales	5.11 ± 1.33	5.46 ± 1.21	6.66 ± 2.00	3.76 ± 0.75	0.91	0.80	0.65
Lachnospirales	3.95 ± 0.70	5.19 ± 0.83	5.70 ± 0.73	5.95 ± 0.86	0.40	0.73	0.73
Coriobacteriales	3.52 ± 0.90^{ab}	5.45 ± 0.55^{a}	5.22 ± 0.85^{a}	1.99 ± 0.28^{b}	0.22	0.36	0.0008
Verrucomicrobiales	4.75 ± 1.42	2.07 ± 0.57	1.05 ± 0.44	7.60 ± 3.92	0.36	0.93	0.76
Oscillospirales	2.71 ± 0.49^{b}	2.51 ± 0.34^{b}	4.49 ± 0.73^{a}	4.57 ± 0.58^{a}	0.0007	0.86	0.85
Clostridia_UCG-014	4.91 ± 1.58	4.37 ± 1.27	0.14 ± 0.01	0.28 ± 0.06	0.35	0.32	0.87
Deferribacterales	0.98 ± 0.57	1.20 ± 0.37	2.16 ± 0.63	2.10 ± 0.60	0.05	0.33	0.22
Burkholderiales	0.18 ± 0.05	0.41 ± 0.10	0.24 ± 0.11	0.34 ± 0.29	0.22	0.86	0.48
Bifidobacteriales	0.70 ± 0.23^{a}	0.40 ± 0.12^{a}	0.17 ± 0.03^{b}	0.09 ± 0.04^{b}	0.0019	0.10	0.33
Peptostreptococcales-Tissierellales	0.48 ± 0.31	0.07 ± 0.01	0.15 ± 0.02	0.20 ± 0.08	0.26	0.24	0.13
Peptococcales	0.10 ± 0.02	0.10 ± 0.01	0.12 ± 0.02	0.14 ± 0.01	0.07	0.55	0.52
**Family**							
Erysipelotrichaceae	36.64 ± 4.02^{b}	33.61 ± 3.21^{b}	48.99 ± 4.02^{a}	41.20 ± 5.42^{a}	0.0230	0.21	0.58
Muribaculaceae	15.79 ± 1.58^{a}	15.05 ± 1.02^{a}	7.88 ± 1.31^{b}	11.24 ± 1.72^{b}	0.0002	0.37	0.16
Desulfovibrionaceae	5.46 ± 0.81	9.95 ± 1.70	9.42 ± 1.79	9.82 ± 2.30	0.28	0.17	0.25
Lachnospiraceae	3.94 ± 0.70	5.19 ± 0.83	5.70 ± 0.73	5.95 ± 0.86	0.40	0.73	0.73

Lactobacillaceae	4.01 ± 1.31	4.62 ± 1.22	4.80 ± 1.82	2.72 ± 0.72	0.83	0.89	0.25
Atopobiaceae	3.46 ± 0.90[ab]	5.41 ± 0.55[a]	5.18 ± 0.85[a]	1.96 ± 0.28[b]	0.22	0.37	0.0007
Akkermansiaceae	4.75 ± 1.42	2.07 ± 0.57	1.05 ± 0.44	7.60 ± 3.92	0.36	0.93	0.76
Clostridia_UCG-014	4.91 ± 1.58	4.37 ± 1.27	0.14 ± 0.01	0.28 ± 0.06	0.35	0.32	0.87
Deferribacteraceae	0.98 ± 0.57	1.20 ± 0.37	2.16 ± 0.63	2.10 ± 0.60	0.05	0.33	0.22
Oscillospiraceae	1.40 ± 0.28[b]	1.57 ± 0.26[b]	1.78 ± 0.17[a]	2.62 ± 0.35[a]	0.0046	0.08	0.57
Bacteroidaceae	3.07 ± 0.43[a]	1.20 ± 0.23[b]	1.44 ± 0.30[b]	0.64 ± 0.15[c]	0.0006	0.0001	0.96
Rikenellaceae	2.78 ± 0.44[a]	2.84 ± 0.40[a]	0.60 ± 0.23[b]	0.89 ± 0.26[b]	0.0229	0.73	0.90
Prevotellaceae	0.88 ± 0.12[a]	1.40 ± 0.35[a]	1.08 ± 0.68[b]	0.51 ± 0.29[b]	0.0012	0.72	0.39
Streptococcaceae	1.29 ± 0.48[a]	0.56 ± 0.08[b]	1.38 ± 0.25[a]	0.53 ± 0.13[b]	0.99	0.0013	0.36
[Eubacterium]_coprostanoligenes_group	0.31 ± 0.09[b]	0.20 ± 0.05[b]	1.77 ± 0.53[a]	0.67 ± 0.28[a]	0.0406	0.10	0.33
Ruminococcaceae	0.62 ± 0.16	0.59 ± 0.10	0.49 ± 0.04	0.63 ± 0.07	0.61	0.35	0.66
Sutterellaceae	0.18 ± 0.05	0.41 ± 0.10	0.24 ± 0.11	0.34 ± 0.29	0.23	0.85	0.48
Bifidobacteriaceae	0.70 ± 0.23[a]	0.40 ± 0.12[a]	0.17 ± 0.03[b]	0.09 ± 0.04[b]	0.0019	0.10	0.33
Aerococcaceae	0.27 ± 0.06[a]	0.18 ± 0.03[b]	0.37 ± 0.08[a]	0.13 ± 0.03[b]	0.71	0.0038	0.18
Peptostreptococcaceae	0.38 ± 0.28	0.02 ± 0.00	0.02 ± 0.00	0.12 ± 0.08	0.44	0.75	0.0226
**Species**							
Mucispirillum_schaedleri	0.98 ± 0.57	1.20 ± 0.37	2.16 ± 0.63	2.06 ± 0.61	0.12	0.52	0.17

[1] Data were presented as mean ± SEM. Statistical analysis was performed using two-way analysis of variance (ANOVA) or Scheirer-Ray-Hare test with the main effects of diet and NR and diet*NR interaction, depending on whether the data were under normal distribution. *P-diet* indicated significant main effect of diet (HFD vs CD), *P-NR* indicated significant main effect of NR supplementation (NR supplementation vs no supplementation), and *P-diet*NR* indicated significant interaction between diet and NR supplementation. Means with different letters (a, b or c) indicated significantly different from each other. CD (control), n = 10; CD + NR, n = 11; CD, control diet; HFD, n = 10; HFD + NR, n = 10. HFD, high-fat and high-cholesterol diet; + NR, a diet plus oral gavage with 0.3 g/kg BW/day of NR.

Supplementary Table S3. Fecal concentrations of short-chain fatty acids in ApoE$^{-/-}$ mice fed with a CD or HFD, without or with NR at the end of the study[1]

Short chain fatty acids (mg/g)	CD	CD + NR	HFD	HFD + NR	*P-diet*	*P-NR*	*P-diet*NR*
Acetic acid	5.53 ± 1.19[a]	7.14 ± 0.67[a]	3.34 ± 0.33[b]	4.04 ± 0.56[b]	0.0127	0.07	0.30
Propionic acid	0.52 ± 0.09[b]	0.88 ± 0.10[a]	0.53 ± 0.06[b]	0.50 ± 0.07[b]	0.0357	0.06	0.0220
Butyric acid	0.54 ± 0.12[a]	0.95 ± 0.20[a]	0.43 ± 0.04[b]	0.43 ± 0.09[b]	0.0225	0.12	0.12
Isobutyric acid	0.08 ± 0.01[b]	0.12 ± 0.01[a]	0.10 ± 0.01[ab]	0.09 ± 0.01[ab]	0.42	0.0342	0.0252
Valeric acid	0.15 ± 0.02	0.19 ± 0.02	0.18 ± 0.01	0.19 ± 0.02	0.51	0.25	0.47
Isovaleric acid	0.05 ± 0.01	0.07 ± 0.00	0.07 ± 0.01	0.05 ± 0.00	0.90	0.95	0.19

[1] Data were presented as mean ± SEM. Statistical analysis was performed using two-way analysis of variance (ANOVA) or Scheirer-Ray-Hare test with the main effects of diet and NR and diet*NR interaction, depending on whether the data were under normal distribution. *P-diet* indicated significant main effect of diet (HFD vs CD), *P-NR* indicated significant main effect of NR supplementation (NR supplementation vs no supplementation), and *P-diet*NR* indicated significant interaction between diet and NR supplementation. Means with different letters (a or b) indicated significantly different from each other. CD (control), n = 10; CD + NR, n = 11; HFD, n = 10; HFD + NR, n = 10. CD, control diet; HFD, high-fat and high-cholesterol diet; + NR, a diet plus oral gavage with 0.3 g/kg BW/day of NR.

Supplementary Table S4. Correlations between relative abundance of gut microbiota and blood biochemical parameters, Oil Red O staining of aorta and gene expression in cholesterol metabolism[1]

	**Blood biochemical parameters**							**Oil Red O staining**				**Hepatic gene expression in cholesterol metabolism**			**Intestinal gene expression in cholesterol metabolism**	
**Taxon**	**TC**	**HDL-C**	**non-HDL-C**	**TC /HDL-C**	**LDL-C /HDL-C**	**Fasting glucose**	**Fasting PCSK9**	**Aorta**	**Aorta arch**	**Thoracic aorta**	**Abdominal aorta**	**HMGCR**	**PCSK9**	**CYP7A1**	**LXR-α**	**ABCA1**
**Phylum**																
Firmicutes	0.46**	N	0.45**	0.36*	0.38*	N	N	N	N	N	0.39*	-0.52***	N	N	N	N
Bacteroidetes	-0.48**	-0.42**	-0.48**	-0.43**	-0.43**	N	N	-0.35*	N	N	-0.34*	0.36*	N	N	N	N
Desulfobacterota	0.48**	N	0.49**	0.48**	0.48**	N	N	0.38*	0.38*	N	N	N	N	N	N	N
Actinobacteria	-0.36*	N	-0.37*	N	N	N	N	-0.47**	-0.43**	-0.42**	N	N	0.43*	N	-0.37*	-0.36*
Verrucomicrobia	-0.56***	N	-0.57***	-0.54***	-0.53***	N	N	-0.41*	N	-0.40*	-0.36*	0.39*	N	N	N	N
Deferribacteres	0.38*	N	0.39*	0.35*	0.36*	N	N	N	N	N	N	N	N	N	N	N
Proteobacteria	-0.54***	N	-0.54***	-0.56***	-0.59***	N	N	-0.39*	N	N	N	0.46**	0.44**	N	N	N
**Class**																
Bacilli	0.45**	0.36*	0.44**	0.33*	0.36*	N	N	N	N	N	0.41*	-0.48**	N	N	N	N
Bacteroidia	-0.48**	-0.42**	-0.48**	-0.43**	-0.43**	N	N	-0.35*	N	N	-0.34*	0.36*	N	N	N	N
Clostridia	N	N	N	N	N	N	N	N	N	N	N	N	N	N	N	N
Desulfovibrionia	0.48**	N	0.49**	0.48**	0.48**	N	N	0.38*	0.38*	N	N	N	N	N	N	N
Coriobacteriia	N	N	N	N	N	N	N	-0.42*	-0.39*	-0.39*	N	N	0.40*	N	-0.37*	-0.40*
Verrucomicrobiae	-0.56***	N	-0.57***	-0.54***	-0.53***	N	N	-0.41*	N	-0.40*	-0.36*	0.39*	N	N	N	N
Deferribacteres	0.38*	N	0.39*	0.35*	0.36*	N	N	N	N	N	N	N	N	N	N	N
Gammaproteobacteria	-0.52***	N	-0.53***	-0.55***	-0.58***	N	N	-0.38*	N	N	N	0.46**	0.43**	N	N	N
Actinobacteria	N	N	N	N	N	-0.39*	N	-0.42**	-0.44**	N	N	N	N	N	N	N
**Order**																
Erysipelotrichales	0.33*	0.34*	0.32*	N	N	N	N	N	N	0.41**	0.36*	-0.55***	N	N	N	N
Bacteroidales	-0.48**	-0.42**	-0.48**	-0.43**	-0.43**	N	N	-0.35*	N	N	-0.34*	0.36*	N	N	N	N
Desulfovibrionales	0.48**	N	0.49**	0.48**	0.48**	N	N	0.38*	0.38*	N	N	N	N	N	N	N
Lactobacillales	N	N	N	N	N	N	N	N	N	N	N	N	N	N	N	N
Lachnospirales	0.39*	N	0.39*	0.32*	N	N	N	N	0.37*	N	N	N	N	N	N	N
Coriobacteriales	N	N	N	N	N	N	N	-0.42*	-0.39*	-0.39*	N	N	0.40*	N	-0.37*	-0.40*
Verrucomicrobiales	-0.56***	N	-0.57***	-0.54***	-0.53***	N	N	-0.41*	N	-0.40*	-0.36*	0.39*	N	N	N	N
Oscillospirales	0.35*	N	0.34*	N	0.32*	N	0.44**	N	0.32*	N	N	N	N	N	N	N

Clostridia_UCG-014	-0.38*	-0.39*	-0.37*	N	N	-0.55***	-0.53***	-0.39*	-0.35*	-0.42**	N	N	N	N	N	N
Deferribacterales	0.38*	N	0.39*	0.35*	0.36*	N	N	N	N	N	N	N	N	N	N	N
Burkholderiales	-0.49**	N	-0.50**	-0.56***	-0.58***	N	N	-0.45**	-0.36*	-0.38*	N	0.46**	0.41*	N	N	N
Bifidobacteriales	N	N	N	N	N	N	N	-0.37*	-0.42**	N	N	N	N	N	N	N
Peptostreptococcales-Tissierellales	0.51**	N	0.52**	0.49**	0.54***	N	N	N	N	N	N	-0.57***	-0.38*	N	N	N
Peptococcales	N	N	N	N	N	N	0.41*	N	0.34*	N	N	N	N	N	N	N
**Family**																
Erysipelotrichaceae	0.33*	0.34*	0.33*	N	N	N	N	N	N	0.41**	0.36*	-0.55***	N	N	N	N
Muribaculaceae	-0.56***	-0.42*	-0.55***	-0.41**	-0.42**	-0.42**	N	-0.35*	N	N	-0.33*	0.44**	N	N	N	N
Desulfovibrionaceae	0.48**	N	0.49**	0.48**	0.48**	N	N	0.38*	0.38*	N	N	N	N	N	N	N
Lachnospiraceae	0.39*	N	0.39*	0.32*	N	N	N	N	0.37*	N	N	N	N	N	N	N
Lactobacillaceae	N	N	N	N	N	N	N	N	N	N	N	N	0.34*	N	-0.33*	N
Atopobiaceae	N	N	N	N	N	N	N	-0.41*	-0.37*	-0.40*	N	N	0.39*	N	-0.37*	-0.40*
Akkermansiaceae	-0.56***	N	-0.57***	-0.54***	-0.53***	N	N	-0.41*	N	-0.40*	-0.36*	0.39*	N	N	N	N
Clostridia_UCG-014	-0.38*	-0.39*	-0.37*	N	N	-0.55***	-0.53***	-0.39*	-0.35*	-0.42**	N	N	N	N	N	N
Deferribacteraceae	0.38*	N	0.39*	0.35*	0.36*	N	N	N	N	N	N	N	N	N	N	N
Oscillospiraceae	0.40*	0.37*	0.41*	0.37*	0.38*	N	N	0.39*	0.56***	N	N	N	N	N	N	N
Rikenellaceae	-0.40*	-0.47**	-0.39*	N	-0.31*	-0.44**	-0.35*	-0.42**	-0.37*	-0.41**	N	0.38*	N	N	N	N
Bacteroidaceae	-0.42**	-0.47**	-0.42**	N	N	N	N	-0.47**	-0.38*	N	-0.35*	N	N	N	N	N
Prevotellaceae	-0.46**	N	-0.46**	-0.40*	-0.38*	N	N	-0.53***	-0.45**	-0.36*	-0.46**	0.32*	N	N	N	N
Streptococcaceae	N	N	N	0.40*	0.44**	N	N	N	N	N	N	-0.47**	N	-0.38*	N	N
[Eubacterium]_coprostanoligenes_group	N	N	N	N	N	0.36*	N	N	N	N	N	N	N	N	N	N
Ruminococcaceae	0.36*	N	0.37*	0.37*	0.37*	N	N	N	0.37*	N	N	N	N	N	N	N
Sutterellaceae	-0.49**	N	-0.49**	-0.56***	-0.58***	N	N	-0.45**	-0.36*	-0.37*	N	0.45**	0.40*	N	N	N
Bifidobacteriaceae	N	N	N	N	N	N	N	-0.37*	-0.42**	N	N	N	N	N	N	N
Aerococcaceae	N	N	N	N	0.34*	N	N	N	N	N	N	-0.51**	N	N	N	N
Peptostreptococcaceae	N	N	N	N	N	N	N	N	N	N	N	N	N	N	N	N
**Genus**																
Faecalibaculum	N	N	N	N	N	N	N	0.38*	N	0.51***	0.42**	-0.54***	-0.38*	N	N	N
Muribaculaceae	-0.57***	-0.44**	-0.56***	-0.40*	-0.41**	-0.45**	N	-0.36*	N	N	N	0.44**	N	N	N	N
Lactobacillus	N	N	N	N	N	N	N	N	N	N	N	N	0.34*	N	-0.33*	N

Dubosiella	0.37*	N	0.37*	0.33*	0.37*	N	N	N	N	N	N	N	N	N	N	N
Coriobacteriaceae_UCG-002	N	N	N	N	N	N	N	-0.41*	-0.37*	-0.40*	N	N	0.39*	N	-0.37*	-0.40*
Akkermansia	-0.56***	N	-0.57***	-0.54***	-0.53***	N	N	-0.41*	N	-0.40*	-0.36*	0.39*	N	N	N	N
Clostridia_UCG-014	-0.38*	-0.39*	-0.37*	N	N	-0.55***	-0.53***	-0.39*	-0.35*	-0.42**	N	N	N	N	N	N
Mucispirillum	0.38*	N	0.39*	0.35*	0.36*	N	N	N	N	N	N	N	N	N	N	N
Alistipes	-0.40*	-0.47**	-0.39*	N	-0.31*	-0.44**	-0.35*	-0.42**	-0.37*	-0.41**	N	0.38*	N	N	N	N
Bacteroides	-0.42**	-0.47**	-0.42**	N	N	N	N	-0.47**	-0.38*	N	-0.35*	N	N	N	N	N
Alloprevotella	-0.46**	N	-0.47**	-0.40*	-0.38*	N	N	-0.54***	-0.47**	-0.37*	-0.46**	0.33*	N	N	N	N
[Eubacterium]_coprostanoligenes_group	N	N	N	N	N	0.36*	N	N	N	N	N	N	N	N	N	N
Lactococcus	N	N	N	0.40*	0.45**	N	N	N	N	N	N	-0.47**	N	-0.37*	N	N
Blautia	0.35*	N	0.35*	N	N	0.37*	0.42*	0.46**	0.51**	N	N	N	N	N	N	N
Lachnospiraceae_NK4A136_group	N	N	N	N	N	-0.35*	N	N	N	-0.41**	N	0.55***	N	N	N	N
Parasutterella	-0.49**	N	-0.49**	-0.56***	-0.58***	N	N	-0.45**	-0.36*	-0.37*	N	0.45**	0.40*	N	N	N
**Species**																
Mucispirillum_schaedleri	0.42**	N	0.42**	0.40*	0.41*	N	N	N	N	N	N	N	N	N	N	N

[1] Pearson or Spearman correlation tests were performed based on whether the data were under normal distribution (N = 41). Data were presented as Pearson or Spearman correlation coefficients. * $P < 0.05$, ** $P < 0.01$, *** $P < 0.001$ and **** $P < 0.0001$ indicated statistically significant correlations. N indicated the correlations were not significant.

Supplementary Table S5. Correlations between relative abundance of gut microbiota and concentrations of short-chain fatty acids in feces[1]

Taxon	Acetic acid	Propionic acid	Isobutyric acid	Butyric acid
**Phylum**				
Firmicutes	-0.43**	-0.36*	N	-0.36*
Bacteroidetes	0.33*	N	N	N
Desulfobacterota	N	-0.39*	N	N
Actinobacteria	0.50**	0.38*	0.34*	N
Verrucomicrobia	0.61****	0.62****	0.34*	0.49**
Deferribacteres	N	N	N	N
Proteobacteria	0.50**	0.42*	N	N
**Class**				
Bacilli	N	N	N	N
Bacteroidia	0.33*	N	N	N
Clostridia	N	N	N	N
Desulfovibrionia	N	-0.39*	N	N
Coriobacteriia	0.48**	0.39*	0.34*	N
Verrucomicrobiae	0.61****	0.62****	0.34*	0.49**
Deferribacteres	N	N	N	N
Gammaproteobacteria	0.50**	0.42*	N	N
Actinobacteria	N	N	N	N
**Order**				
Erysipelotrichales	N	N	N	N
Bacteroidales	0.33*	N	N	N
Desulfovibrionales	N	-0.39*	N	N
Lactobacillales	N	N	N	-0.34*
Lachnospirales	-0.38*	N	N	N
Coriobacteriales	0.48**	0.39*	0.34*	N
Verrucomicrobiales	0.61****	0.62****	0.34*	0.49**
Oscillospirales	-0.53***	-0.43**	-0.35*	-0.37*
Clostridia_UCG-014	0.34*	N	N	N
Deferribacterales	N	N	N	N
Burkholderiales	0.63****	0.55***	N	0.43**
Bifidobacteriales	N	N	N	N
Peptostreptococcales-Tissierellales	-0.78****	-0.63****	-0.49**	-0.53***
Peptococcales	-0.37*	N	-0.39*	N
**Family**				
Erysipelotrichaceae	N	N	N	N
Muribaculaceae	0.34*	N	N	N
Desulfovibrionaceae	N	-0.39*	N	N
Lachnospiraceae	-0.38*	N	N	N
Lactobacillaceae	N	N	N	N
Atopobiaceae	0.48**	0.39*	0.34*	N
Akkermansiaceae	0.61****	0.62****	0.34*	0.49**
Clostridia_UCG-014	0.34*	N	N	N
Deferribacteraceae	N	N	N	N
Oscillospiraceae	-0.43**	-0.49**	-0.35*	-0.37*
Rikenellaceae	0.49**	N	N	0.34*
Bacteroidaceae	N	N	-0.37*	N
Prevotellaceae	0.48**	0.32*	N	0.36*
Streptococcaceae	-0.51**	-0.44**	N	-0.32*
[Eubacterium]_coprostanoligenes_group	N	N	N	N

Ruminococcaceae	N	-0.41*	N	-0.34*
Sutterellaceae	0.63****	0.55***	N	0.43**
Bifidobacteriaceae	N	N	N	N
Aerococcaceae	N	N	N	N
Peptostreptococcaceae	N	-0.36*	N	-0.45**
**Genus**				
Faecalibaculum	N	N	N	N
Muribaculaceae	0.34*	N	N	N
Lactobacillus	N	N	N	N
Dubosiella	N	N	N	-0.35*
Coriobacteriaceae_UCG-002	0.48**	0.39*	0.34*	N
Akkermansia	0.61****	0.62****	0.34*	0.49**
Clostridia_UCG-014	0.34*	N	N	N
Mucispirillum	N	N	N	N
Alistipes	0.49**	N	N	0.34*
Bacteroides	N	N	-0.37*	N
Alloprevotella	0.48**	0.34*	N	0.34*
[Eubacterium]_coprostanoligenes_group	N	N	N	N
Lactococcus	-0.52***	-0.44**	N	-0.33*
Blautia	-0.48**	N	N	N
Lachnospiraceae_NK4A136_group	N	N	N	N
Parasutterella	0.63****	0.55***	N	0.43**
**Species**				
Mucispirillum_schaedleri	N	N	N	N

[1] Pearson or Spearman correlation tests were performed based on whether the data were under normal distribution (N = 41). Data were presented as Pearson or Spearman correlation coefficients. * $P < 0.05$, ** $P < 0.01$, *** $P < 0.001$ and **** $P < 0.0001$ indicated statistically significant correlations. N indicated the correlations were not significant.
